# The spinal cord is never at rest

**DOI:** 10.7554/eLife.03811

**Published:** 2014-08-05

**Authors:** Falk Eippert, Irene Tracey

**Affiliations:** 1 **Falk Eippert** is in the Oxford Centre for Functional Magnetic Resonance Imaging of the Brain, Nuffield Department of Clinical Neurosciences, University of Oxford, Oxford, United Kingdomfalk.eippert@ndcn.ox.ac.uk; 2 **Irene Tracey** is in the Oxford Centre for Functional Magnetic Resonance Imaging of the Brain, Nuffield Department of Clinical Neurosciences and Nuffield Division of Anaesthetics, University of Oxford, Oxford, United Kingdomirene.tracey@ndcn.ox.ac.uk

**Keywords:** fMRI, spinal cord, 7 Tesla, resting state, functional connectivity, human

## Abstract

Even when we are at rest, our spinal cords show spontaneous, yet well organised, fluctuations of activity that might reflect sensory and motor networks.

**Related research article** Barry RL, Smith SA, Dula AN, Gore JC. 2014. Resting state functional connectivity in the human spinal cord. *eLife* **3**:e02812. doi: 10.7554/eLife.02812

**Image** The levels of activity in different parts of the spinal cord of resting individuals were measured using fMRI

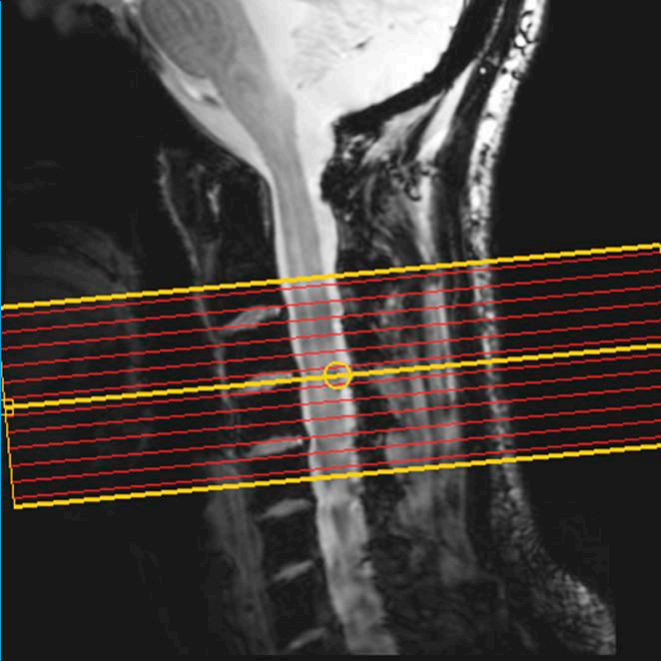

Even when we are resting, our brain is far from inactive and instead displays an enormous amount of spontaneous activity. This activity is not just noise, but is organised in both space and time, with different brain networks showing distinct fluctuations. Moreover, these ‘resting-state signals’ account for most of the energy consumed by the brain.

Resting-state signals are organised into several distinct networks that can be modified by experience and can also change in response to numerous diseases, but it is still unclear what causes these signals (Fox and Raichle, 2007; Buckner et al., 2013). Now, in *eLife*, Robert Barry of Vanderbilt University and co-workers—including Seth Smith, Adrienne Dula and John Gore—report that such resting-state signals can also be seen in the human spinal cord (Barry et al., 2014). Thus, these signals seem to be a hallmark of the entire central nervous system.

The spinal cord is the brain's main interface with the rest of the body. The centre of the spinal cord is made up of four ‘horns’. The two dorsal horns at the back of the spinal cord contain the neurons that receive sensory information from the body; and the two ventral horns at the front contain the neurons that send signals to the muscles. The Vanderbilt team recorded activity from the human spinal cord using a technique called fMRI (short for functional magnetic resonance imaging). fMRI can measure neuronal activity indirectly by detecting changes in blood flow: when an area of the brain is in use, blood flow to that region increases (Logothetis, 2008). It is also non-invasive as it only involves positioning a person inside an MRI scanner.

Performing fMRI of the spinal cord, however, is technically challenging for a number of reasons. In particular, the spinal cord is very small (around 12 mm in diameter; Figure 1A), and there are also various sources of noise that degrade the quality of the signal to a much greater degree than happens in brain imaging (Summers et al., 2014). Correspondingly, the first reports of spinal fMRI only occurred in the late 1990s (Yoshizawa et al., 1996; Stroman et al., 1999) and relatively few groups are currently undertaking such studies.

**Figure 1.**
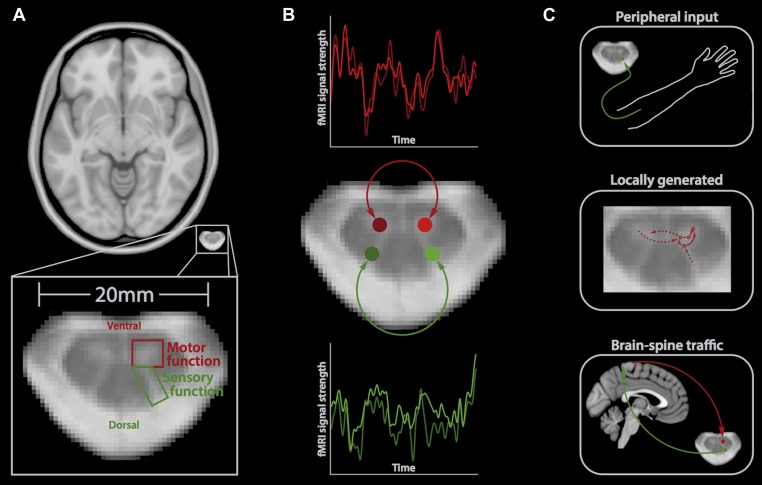
Resting-state signals in the human spinal cord. (**A**) Horizontal section of a brain (top) and a spinal cord (middle, bottom); the small size of the spinal cord makes it difficult to image neuronal activity. The spinal cord contains two ventral horns (one outlined in red) that are involved in motor function, and two dorsal horns (one outlined in green) that are involved in sensory function. (**B**) Barry et al. measured the correlation between spontaneous fluctuations in the fMRI signal in the ventral horns (red traces; top) and the dorsal horns (green traces; bottom). This revealed that the ventral horns show a positive correlation with each other, as do the dorsal horns. However, there is no significant correlation between ventral and dorsal horns. This suggests that at rest, the spinal cord is intrinsically organised into two separate networks, corresponding to motor and sensory functions. (**C**) Possible mechanisms that could explain the spontaneous activity in the spinal cord include input from the peripheral nervous system (top), locally generated rhythms from the interneurons within spinal networks (middle), and ongoing communication between the brain and spinal cord (bottom).

Barry et al. took the level of technical difficulty one step further by imaging the spinal cord at a very high magnetic field strength. Although this exacerbates many of the problems in spinal fMRI (problems that were overcome by developing tailored data acquisition and analysis techniques), it also provides a unique opportunity to look at the spinal cord in very fine detail and with a high signal-to-noise ratio. Barry et al. acquired fMRI data from the spinal cords of a group of healthy volunteers, who were simply resting inside the MRI scanner. Next, they extracted the fMRI signal over time from both ventral horns and both dorsal horns, and investigated the correlation between the activity in each of these four regions over time (Figure 1B).

Barry et al. found highly significant positive correlations between the left and right ventral (‘motor’) horns, and also between the left and right dorsal (‘sensory’) horns, suggesting that these structures form two distinct networks. This is obviously in good agreement with the functional organisation of the spinal cord. It also, to some degree, reflects the patterns of spontaneous fluctuations in the brain, where distinct resting-state networks (e.g., visual, auditory, etc) are observed (Fox and Raichle, 2007).

Barry et al. did not observe significant correlations in activity between the ventral and dorsal horns, suggesting that although the connections between these structures are essential for reflex responses, they might otherwise be dormant. However, it remains to be tested if the correlations in the spontaneous fluctuations will continue when an individual is performing a task or whether different patterns will emerge (Cole et al., 2014).

It is natural to assume that the observed resting-state correlations of fMRI signals reflect communication or connections between neurons. However, a variety of other mechanisms that are not related to neuronal activity are known to contribute to resting-state fMRI signals, including breathing-related changes and pulsations that are caused by each heartbeat (Birn, 2012). Barry et al. used several different techniques to address these issues, which strengthens the idea that their results do indeed reflect the activity of the spinal cord at rest.

So, what is the mechanism behind the observed spontaneous fluctuations in the spinal cord? There are several possible answers to this question, which are not mutually exclusive (Figure 1C). First, these fluctuations could be driven by ongoing input from the peripheral nervous system relaying information to the central nervous system, for example, about the relative position of body parts. Second, they could reflect signals that are generated locally within the spinal cord, including those created by the central pattern generators that control activities such as walking (Grillner and Jessell, 2009). Third, the fluctuations could reflect ongoing sensory and motor signals travelling up and down between the brain and spinal cord. Most likely, a mixture of these (and possibly other) mechanisms is involved, and it will require carefully designed experiments to disentangle these mechanisms.

In any case, the possibility to non-invasively investigate if the spinal cord's sensory and motor circuits are functioning properly is of enormous clinical interest. Many neurological disorders involve the spinal cord and the most obvious example is spinal cord injury. In these instances, the method developed by the Vanderbilt team could complement approaches that are focussed on structural changes (Freund et al., 2013). Yet, for this to happen we first need to answer several other questions: what is the organisation of connectivity across the different segments of the spine? Are the reported networks stable over time or do they change? Also, how do the different networks (sensory and motor) interact with each other? Finally, it would be interesting to record such resting-state signals from the brain and spinal cord at the same time (Finsterbusch et al., 2013) in order to obtain an integrative picture of the function of the central nervous system.
